# (I Can’t Get No) Saturation: A simulation and guidelines for sample sizes in qualitative research

**DOI:** 10.1371/journal.pone.0181689

**Published:** 2017-07-26

**Authors:** Frank J. van Rijnsoever

**Affiliations:** 1 Innovation Studies, Copernicus Institute of Sustainable Development, Utrecht University, Utrecht, The Netherlands; 2 INGENIO (CSIC-UPV), Universitat Politècnica de València, Valencia, Spain; Lancaster University, UNITED KINGDOM

## Abstract

I explore *the sample size in qualitative research that is required to reach theoretical saturation*. I conceptualize a population as consisting of sub-populations that contain different types of information sources that hold a number of codes. Theoretical saturation is reached after all the codes in the population have been observed once in the sample. I delineate three different scenarios to sample information sources: “random chance,” which is based on probability sampling, “minimal information,” which yields at least one new code per sampling step, and “maximum information,” which yields the largest number of new codes per sampling step. Next, I use simulations to assess the minimum sample size for each scenario for systematically varying hypothetical populations. I show that theoretical saturation is more dependent on the mean probability of observing codes than on the number of codes in a population. Moreover, the minimal and maximal information scenarios are significantly more efficient than random chance, but yield fewer repetitions per code to validate the findings. I formulate guidelines for purposive sampling and recommend that researchers follow a minimum information scenario.

## Introduction

Qualitative research is becoming an increasingly prominent way to conduct scientific research in business, management, and organization studies [1]. In the first decade of the twenty-first century, more qualitative research has been published in top American management journals than in the preceding 20 years [2]. Qualitative research is seen as crucial in the process of building new theories [2–4] and it allows researchers to describe how change processes unfold over time [5,6]. Moreover, it gives close-up and in-depth insights into various organizational phenomena [7,8] perspectives and motivations for actions [1,8]. However, despite the explicit attention of journal editors to what qualitative research is and how it could or should be conducted [8–10], it is not always transparent how particular research was actually conducted [2,11]. A typical topic of debate is what the size of a sample should be for inductive qualitative research to be credible and dependable [9,12] (Note that in this paper, I refer to qualitative research in an inductive context. I recognize that there are more deductive-oriented forms of qualitative research).

A general statement from inductive qualitative research about sample size is that the data collection and analysis should continue until the point at which no new codes or concepts emerge [13,14]. This does not only mean that no new stories emerge, but also that no new codes that signify new properties of uncovered patterns emerge [15]. At this point, “theoretical saturation” is reached; all the relevant information that is needed to gain complete insights into a topic has been found [1,13]. (Note that to prevent confusion, I use the term ‘code’ in this article to refer to information uncovered in qualitative research. I reserve the term ‘concept’ to refer to the concepts in the theoretical framework).

Most qualitative researchers who aim for theoretical saturation do not rely on probability sampling. Rather, the sampling procedure is purposive [14,16]. It aims “to select information-rich cases whose study will illuminate the questions under study” [12]. The researcher decides which cases to include in the sample based on prior information like theory or insights gained during the data collection.

However, the minimum size of a purposive sample needed to reach theoretical saturation is difficult to estimate [9,17–22].

There are two reasons why the minimum size of a purposive sample deserves more attention. First, theoretical saturation seems to call for a “more is better” sampling approach, as this minimizes the chances of codes being missed. However, the coding process in qualitative research is laborious and time consuming. As such, especially researchers with scarce resources do not want to oversample too much. Some scholars give tentative indications of sample sizes that often lie between 20 and 30 and are usually below 50 [23,24], but the theoretical mechanism on which these estimates are based is unknown.

Second, most research argues that determining whether theoretical saturation has been reached remains at the discretion of the researcher, who uses her or his own judgment and experience [9,22,25,26]. Patton [12] even states that “there are no rules for sample size in qualitative inquiry” (p. 184). As such, the guidelines for judging the sample size are often implicit. The reason for this is that most qualitative research is largely an interpretivistic endeavor [27] that requires flexible creative thinking, experience, and tacit knowledge [9]. However, researchers from the field of management [8,11,28], information sciences [24,29], health [30,31] and the social sciences in general [12,13,32,33], acknowledge the need for transparency in the process of qualitative research. Moreover, not all researchers have the required experience to assess intuitively whether theoretical saturation has been reached. For them, articulating the assessment criteria in a set of guidelines can be helpful [33].

In this paper I explore the sample size that is required to reach theoretical saturation in various scenarios and I use these insights to formulate guidelines about purposive sampling. Following a simulation approach, I assess experimentally the effects of different population parameters on the minimum sample size. I first generate a series of systematically varying hypothetical populations. For each population, I assess the minimum sample sizes required to reach theoretical saturation for three different sampling scenarios: “random chance,” which is based on probability sampling, “minimal information,” which yields at least one new code per sampling step, and “maximum information,” which yields the largest number of new codes per sampling step. The latter two are purposive sampling scenarios.

The results demonstrate that theoretical saturation is more dependent on the mean probability of observing codes than on the number of codes in a population. Moreover, when the mean probability of observing codes is low, the minimal information and maximum information scenarios are much more efficient in reaching theoretical saturation than the random chance scenario. However, the purposive scenarios yield significantly fewer multiple observations per code that can be used to validate the findings.

By using simulations, this study adds to earlier studies that base their sample size estimates on empirical data [16,17], or own experience [22]. Simulating the factors that influence the minimum purposive sample size gives these estimates a theoretical basis [34]. Moreover, the simulations show that the earlier empirical estimates for theoretical saturation are reasonable under most purposive sampling conditions. To my knowledge, there is one earlier study that uses simulations to predict minimum sample size in qualitative research based on random sampling [35]. The present study extends this work by taking into account the process of purposive sampling, using different sampling scenarios.

Based on my analyses, I offer a set of guidelines that researchers can use to estimate whether theoretical saturation has been reached. These guidelines help to make more informed choices for sampling and add to the transparency of the research, but are by no means intended as mechanistic rules that reduce the flexibility of the researcher [10].

In section 2, I discuss the theoretical concepts about purposive sampling. Section 3 describes the simulation, and the results are presented in section 4. In section 5, I draw conclusions, discuss the limitations, and offer recommendations.

## Theoretical concepts

I base this section largely on the existing literature on purposive sampling. I also introduce some new ideas that are sometimes implied by the literature, but that were never conceptualized. Table 1 summarizes the main concepts in this paper, and the symbols used to denote them.

**Table 1:** An overview of the main concepts, definitions and symbols.

Concept	Definition	Symbol
Information source	The unit from which information is gathered	*i*
Population	The total set of information sources that are potentially relevant to answering the research question	*J*
Sub-population	A subset of information sources that are potentially relevant to answering the research question	*j*
Sampling step	The number of information sources sampled so far	*n*
Code	A unique piece of information in the population relevant to the research	*C*_*k*_
Number of codes	The number of unique pieces of information relevant to the research in the population	*k*
Theoretical saturation	All codes are observed at least once.	*s*
Probability of reaching theoretical saturation	The probability that each code is observed at least once	*p*_*n*_
Sampling steps to reach theoretical saturation	The number of sampling steps needed to observe each code at least once	*n*_*s*_
Mean probability of observing codes	The mean probability that a code is observed at an information source	*$\bar{\Phi_{c}}$*
Repetitive codes	Codes that are observed more than once.	-
Minimum number of repetitive codes	The minimum number of times that a code needs to be observed	*v*
Sampling strategy	How the researcher selects the information sources; commonly empirically based.	-
Sampling scenario	Three theory based scenarios on how the sampling process proceeds: random chance, minimal information, maximal information	-
Efficiency	The fewer sampling steps that a scenario requires to reach theoretical saturation, the more efficient it is	-

### Populations, information sources, and sampling steps

A population is the “universe of units of analysis” from which a sample can be drawn [36]. However, in qualitative research, the unit of analysis does not have to be the same as the unit from which information is gathered. I call the latter “information sources.” In the context of interviews, information sources are often referred to as informants [16,37], but they can be any source that informs the researcher: other examples are sites to collect observational data, existing documents, or archival data. I refer to the total set of information sources that are potentially relevant to answering the research question as the population.

From this population, one or multiple information sources are sampled as part of an iterative process that includes data collection, analysis, and interpretation. At each iteration the researcher has the opportunity to adjust the sampling procedure and to select a new information source to be sampled. I assume in this paper that at each iteration only one source is sampled; this assumption has no further consequences for the remainder of the paper. Moreover, I use the term “sampling steps” rather than iterations, as this excludes analysis and interpretation. Finally, contrarily to formal quantitative sampling terminology, I count as sampling steps only observations that participated in the research, thus excluding non-response or the inability to access sources. This eases interpretation.

### Sub-populations

A population of information sources is usually not homogeneous. Multiple *sub-populations* can often be distinguished, for example the difference between interviewees, documents, or focus groups. This is important as the researcher can choose different sampling procedures and data collection methods for each sub-population. The exact delineation of sub-populations depends on the judgment of the researcher. However, I argue there are a number of restrictions on the delineation of sub-populations.

- First, if there are differences in the type of information source, sampling strategy, type of data, data collection, or methods of analysis, then there are sub-populations. The reason for this criterion is that different methods are needed. These different methods need to be accounted for [32] as they can explain differences in outcomes.
- Second, information sources should be interchangeable at the sub-population level. Within a sub-population, no single information source may be critical for reaching theoretical saturation. Hence, no single information source in a sub-population can contain information that is not found in other information sources in that sub-population. The reason for this criterion is that if a particular information source is critical for theoretical saturation, it should by definition be included in the research. Observing critical information is not guaranteed if the inclusion is dependent on a particular sampling strategy. A critical information source should then be treated as a separate sub-population of size one.
- Second, if cases or groups are compared, it is important to treat these as sub-populations. For example, distinguishing between sub-populations is a condition for data triangulation, because the researcher effectively compares the results from one sub-population (for example interviews with managers) with the results from another (for example annual reports). Furthermore, comparative case studies [4,38] involve the comparison of sub-populations.

The concept of sub-populations implies that theoretical saturation can be reached at the level of the overall population or at the level of the sub-population. Reaching theoretical saturation in all the sub-populations is not a condition for reaching theoretical saturation at the level of the population, since sub-populations can have an overlap in information. However, it is necessary to reach theoretical saturation in each sub-population in comparative research or when triangulating results, as this is the only way to make a valid comparison.

### Codes and theoretical saturation

In most cases of inductive qualitative research, information is extracted from information sources, interpreted and translated into codes. I refer to codes here in the context of inductive qualitative data analysis, which means that they can be seen as “tags” or “labels” on unique pieces of information [13]. Codes can represent any sort of information and may be related to each other (for example phenomena, explanations or contextualization). The only conditions that I impose are that each code represents only one piece of information and that two different codes are not allowed to represent the same information. In practice, this means that synonyms are removed during qualitative data analysis. Thereby, codes can be interpreted as unique “bits” of information.

The population contains all the codes that can be potentially observed. At the start of a study, the codes in the population are unobserved and the exact number of codes in the population is unknown. Consulting information sources sampled from the population allows codes to become observed. Theoretical saturation is reached when each code in the population has been observed at least once.

### Number of codes and mean probability of observing codes

I let, the number of sampling steps required to reach theoretical saturation depend on two population characteristics. First, the larger the *number of codes* distinguished in the population, the more sampling steps are required to observe them all. The number of codes can vary greatly per study, depending on the complexity of the research question, and the amount of theory in the literature. A number of 100 is common. Second, the more often a code is present in the population, the larger are the chances that it will become observed. As theoretical saturation takes place at the population level, the distribution of codes in the population is important. For example, interviews can vary in length or some documents can contain more relevant information than others. In general, one would expect that the higher the “mean probability of observing codes” in a population is, the fewer sampling steps are required to reach theoretical saturation. By definition, these probabilities vary between 0 and 1. A mean probability of observing codes of 0.5 means that, on average, a code is observed at 50% of the information sources.

Purposive sampling allows the researcher to make an informed estimation about the probability of observing a given code at each sampling step, using (theoretical) prior information, like sampling frames [39] or insights gained during the data analysis. (This conceptualizing of purposive sampling is also consistent with the notion of theoretical sampling. Both terms are often used interchangeably. Theoretical sampling can be seen as a special case of purposive sampling [14]). However, when the number of codes is large, it is easier simply to estimate the mean probability of observing all the codes in the population. To make such estimations, it is important to consider what the probability of observing codes actually represents. The probability of a code being present at least depends on: the likelihood of an information source actually containing the code, the willingness and ability of the source (or its authors) to let the code be uncovered, and the ability of the researcher to observe the code. These probability estimations are based on the characteristics of the information source and the researcher. The probability of observing a certain code can decrease when the information source (for example an interviewee) has strategic reasons not to share information. The strategic behavior of actors can also lead to the discovery of other additional codes about the motivations and the actions of these actors. The relevance of these codes depends on the research question. In addition, if the researcher has less experience with the technique used to uncover codes from a source or with correctly interpreting information during the data analysis, the probability of observing codes decreases. Having multiple independent coders, on the other hand, can increase the probability of observing a code.

### Repetitive codes

Some researchers consider codes that are observed more than once as redundant, since they do not add new information to the data [12,26,32]. I refer to codes that are observed more than once more neutrally as “repetitive codes.” Repetitive codes are important for a methodological purpose: they can help guard against misinformation. That is, information sources may have given false codes, for reasons of social desirability, strategy, or accidental errors.

To guard against misinformation and to enhance the credibility of the research, it can be advisable to aim for a sample in which each code is observed multiple times (this also follows from the logic behind triangulation). One could argue that if a code, after a substantial number of sampling steps, is still observed only once while almost all other codes have a higher incidence, a critical examination of the code is warranted. In many cases, the researcher may already be suspicious of such a code during the analysis. A frequency of one does not mean that the code is wrong by definition; it is possible that the code is just rare or that the low frequency is just a coincidence. However, it is relatively easy to make an argumentative judgment about the plausibility of rare codes (for example based on theory).

### Sampling strategies, sampling scenarios, and efficiency

A sampling strategy describes how the researcher selects the information sources. The most elaborate inventory of sampling strategies comes from Patton [12], who identifies 15 purposive sampling strategies for qualitative research. Examples include “maximum variation sampling,” “typical case sampling,” and “snowball sampling”. These strategies are based strongly on research practices, but the underlying theoretical criteria for distinguishing between the strategies are left implicit. For example, a criterion that can explain the difference between “maximum variation sampling,” “typical case sampling,” and “extreme case sampling” is the focus of the research question. “Snowball sampling” and “opportunistic sampling” differ in the way in which they obtain information about the next information source that is to be sampled. “Confirming or disconfirming sampling” and “including politically sensitive cases” as strategies are motivated by a delineation of the population. Overall, Patton [12] acknowledges that purposive sampling in qualitative research can be a mixture of the strategies identified and that some of these strategies overlap. These strategies also make implicit assumptions regarding the prior knowledge of the researcher about the population. For example, “extreme case sampling” implicitly assumes that the researcher has knowledge about the full population; otherwise, he or she would be not be able to identify the extreme cases. “Snowball sampling” assumes that the researcher does not have full knowledge of the population, as relevant leads are only identified at each sampling step.

I use the concepts described above to formulate three generic sampling scenarios. I refer to sampling scenarios to avoid confusion with the sampling strategies. The term scenarios term signifies that they are based on theoretical notions, instead of empirical data or observed practices. The three sampling scenarios are based on the number of newly observed codes that a sampled information source adds. This criterion is motivated by the premise of purposive sampling: based on the expected information, the researcher makes an informed decision about the next information source to be sampled at each sampling step. This informed decision implies that the researcher can thus reasonably foresee whether, and perhaps how many, new codes will be observed at the next sampling step. The fewer sampling steps that a scenario requires to reach theoretical saturation, the more efficient it is.

The three scenarios that I identify are “random chance,” “minimal information,” and “maximal information.”

- **Random chance** assumes that the researcher does not use prior information during each sampling step. The researcher randomly samples an information source from the population and adds it to the sample. This scenario is solely based on probability and is considered to be inappropriate for most qualitative studies [14,16]. However, there are good reasons to include this scenario. First, there are conditions under which random chance is an appropriate scenario for sampling. One of these is when no information is gained about the population during the sampling steps, such as when documents or websites are analyzed. Second, random chance can be seen as a worst-case scenario. If a researcher is uncertain about how a sampling process actually worked, it is always possible to explore whether theoretical saturation would have been reached under the conservative conditions of random chance. Third, random chance is the only scenario for which the number of sampling steps can be calculated mathematically. Finally, the random chance scenario can serve as a benchmark to which the number of sampling steps in the other scenarios can be compared.
- **Minimal information** is a purposive scenario that works in the same way as random chance, but adds as extra condition that at least one new code must be observed at each sampling step. This is equivalent to a situation in which the researcher actively seeks information sources that reveal new codes, for example by making enquiries about the source beforehand. It is not uncommon for a researcher to discuss topics with a potential interviewee prior to the actual interview to assess whether the interview will be worthwhile. The minimal information scenario captures these kinds of enquiries. Similarly, researchers may be referred to a next source that adds new codes as part of a snowball strategy. Overall, the criterion of observing at least one new code per sampling step seems to be relatively easy to achieve as long as the researcher has some information about the population at each step. This makes the scenario broadly applicable and more efficient than random chance.
- **Maximal information** is a purposive scenario that assumes that the researcher has almost full knowledge of the codes that exist in the population and the information sources in which they are present. At each sampling step, an information source is added to the sample that leads to the largest possible increase in observed codes. This scenario is in line with the theoretical aim of purposive sampling. However, it does not reflect scenarios where populations' sizes are unknown and too large. It makes large assumptions regarding the prior knowledge of the researcher about the population. An example of when this scenario might be realistic occurs when the researcher is extremely familiar with the field and the specific setting that he or she is investigating.

## Simulation

I use simulations as they allow me to assess the effects of the three scenarios for a series of hypothetical populations that vary systematically regarding (1) the number of codes in the population and (2) the mean probability of observing codes. The controlled setting allows me to assess the relative influence of each of these factors on the reaching of theoretical saturation. In an empirical setting, this would not be possible, because the researcher can generally not control the characteristics of the population under study, because the number of populations that can be studied is limited and because it is never entirely certain whether theoretical saturation has been reached [27].

To keep the paper readable for audiences with either a quantitative or qualitative background, I minimize the mathematical details in the main text as much as possible. The full technical details of the simulation are in S1 Appendix, which can be read instead of sections 3.1 and 3.2. To relate sections 3.1 and 3.2 to S1 Appendix, I assign symbols to the most important concepts in the main text, and refer to the appropriate sections of S1 Appendix.

### Definitions

I denote the number of sampling steps to reach theoretical saturation by *n*_*s*_, and the number of codes in the population as *k*. Theoretical saturation is reached when all *k* codes are observed (see S1 Appendix Section A: Definitions). I further denote the mean probability of observing codes as $\bar{\Phi_{c}}$. I take the mean, because not all codes have the same probability of being observed. This means that some codes are more difficult to uncover than others (see S1 Appendix Section B: Mean probability of observing codes). However, making the unrealistic assumption that all codes have the same probability of being uncovered allows me to calculate the number of sampling steps mathematically (see S1 Appendix Section C: Reaching theoretical saturation). This calculation is not a result of the paper, it only helps me to validate results from the simulations. When there is a difference in probabilities of observing codes, it is not possible to mathematically calculate the number of sampling steps. Therefore, I use simulations. I denote the required minimum number of occurrences of a code by *v*. I will calculate the effect of this factor on the number of sampling steps for theoretical saturation (see S1 Appendix Section D: Repetitive codes). Finally, my simulations apply to the sub-population level, the results for the sub-populations can be aggregated to the population level (see S1 Appendix Section E: From sub-population to population).

### Simulation of scenarios

Using the R-program [40], I generate 1100 hypothetical populations of 5000 information sources. The populations vary systematically by the number of codes *(k)* from 1 to 101 with increments of 10. I let the mean probability of observing codes vary between 0.09 (1/11) and 0.91 (10/11) (see S1 Appendix Section F: Simulation). Further, in line with my earlier argument about interchangeability of information sources, I impose a condition whereby each code should actually be present in at least two information sources in the population.

**Fig 1:**
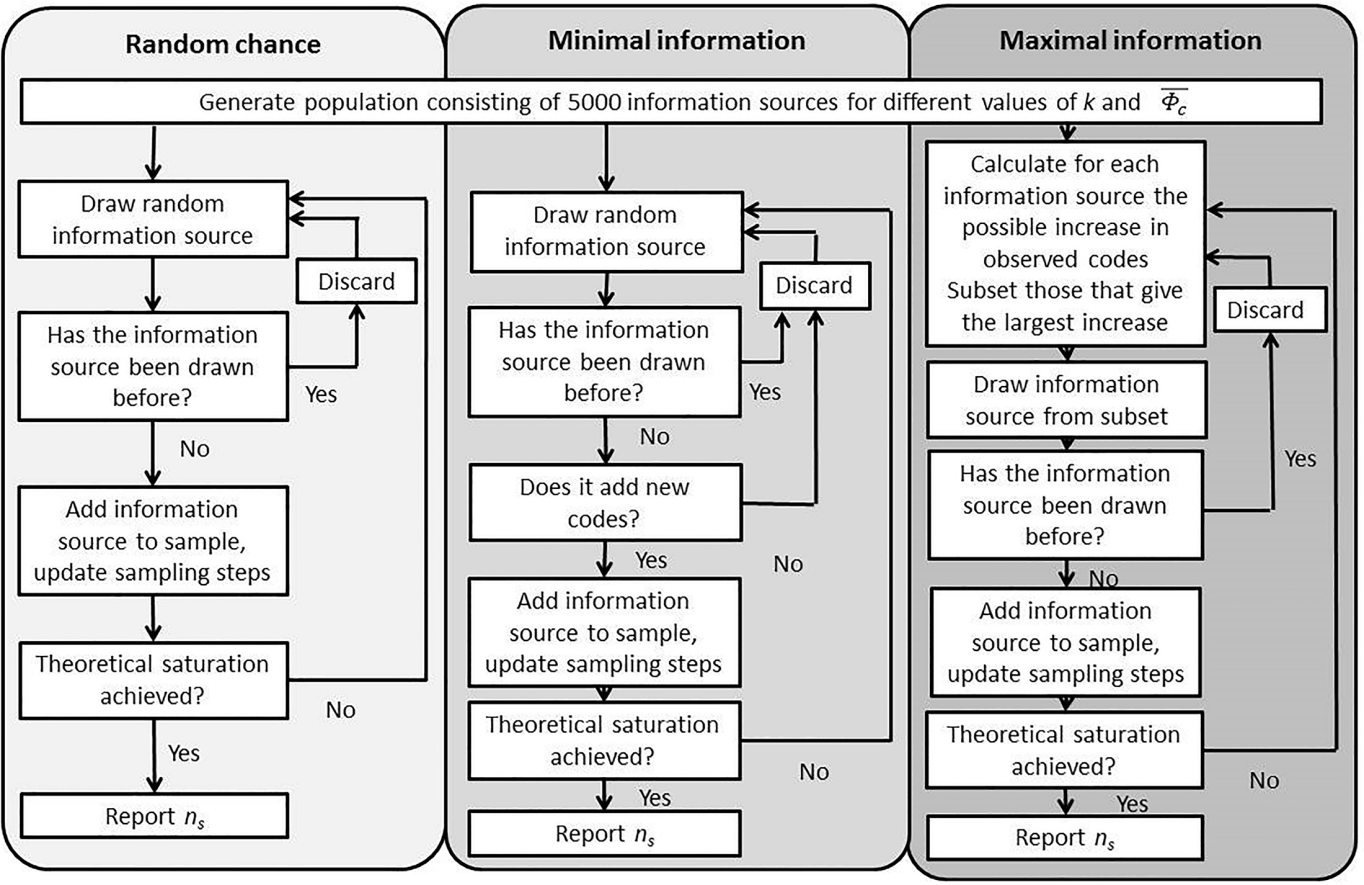
A schematic overview of how the algorithms for each scenario operate.

For each hypothetical population, I simulate the number of sampling steps necessary to reach theoretical saturation under the three scenarios from section 2.5. Fig 1 gives a schematic overview of how the algorithms for each scenario operate. The full R-code is available as S1 File: R-code for the simulations, the resulting data is available as S2 File: Simulated data.

All three scenarios operate in a similar manner. After generating a population, an information source is selected:

- **Random chance** selects information sources based on probability.
- **Minimal information** works in the same way as random chance, but adds as extra condition that at least one new code must be observed per sampling step. Otherwise the information source is discarded, and does not count towards the number of sampling steps.
- **Maximal information** first identifies a set of information sources that contain the largest number of unobserved codes. From this set, which often consists of a small number of information sources, it randomly selects an information source.

If the source has not been selected before, it is added to the sample. After each sampling step, the model evaluates if theoretical saturation is reached. If so, the process stops and the number of sampling steps *n*_*s*_ is reported. Otherwise, the next sampling step takes place and a new information source is selected from the population.

As there are multiple combinations of information sources that allow reaching theoretical saturation per population, I apply each of the three sampling scenarios to each population 500 times. This produces a distribution for each scenario with values of the number of sampling steps to reach theoretical saturation. From this distribution, I report the value that leads to theoretical saturation in 95% of the 500 simulations of a population as main outcome. The value of 95% is in line with statistical conventions, and makes my results more robust.

Finally, for each code in a population, I calculate the mean number of occurrences over the 500 simulations. From this set of numbers I again take the mean, which I denote by $\bar{F}$. This number serves as an indicator of repetitive codes

## Results

**Fig 2:**
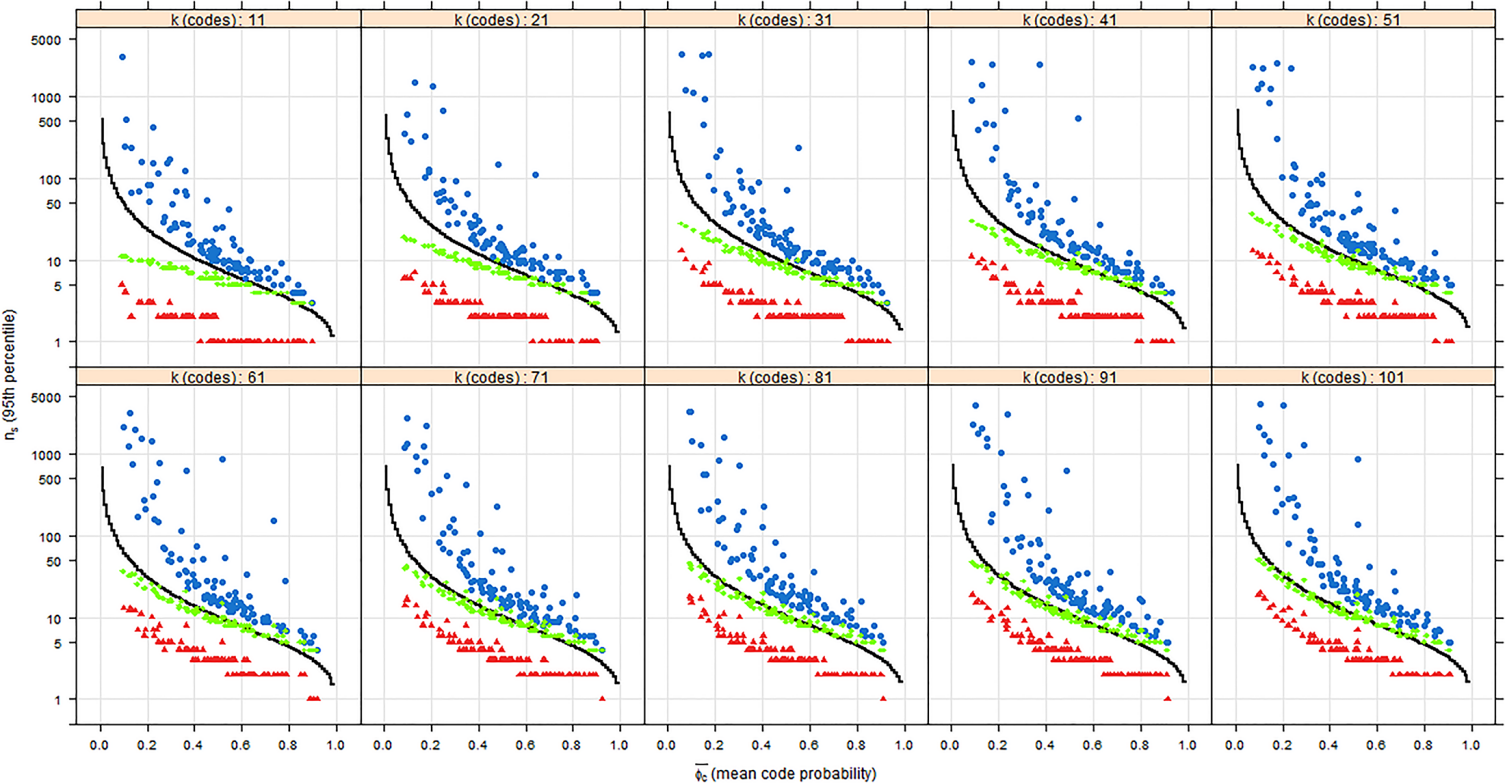
The 95^th^ percentile of *n*_*s*_ against $\bar{\Phi_{c}}$ for the values of k between 11 and 101. Note that the y-axis is logarithmic. The solid black line indicates the calculated random chance’s value of *n* based on *F11*. The blue dots represent random chance, the green diamonds represent minimal information, and the red triangles represent maximal information.

Fig 2 plots the 95^th^ percentile of the number of sampling steps required to reach theoretical saturation *(n*_*s*_*)* against the mean probability of observing codes $\left(\bar{\Phi_{c}}\right)$ for the number of codes in the population (*k)*. Note that the y-axis is logarithmic. The solid black line indicates the mathematically calculated values based on random chance, and no variation in probabilities of observing codes. The dots represent the simulated random chance scenario, the diamonds represent minimal information, and the triangles represent maximal information. The random chance scenario generally follows the mathematical calculation based on random chance, but requires more sampling steps. This is due to the fact that having a variation in the mean probability of observing codes, means that some codes become rare, which requires more sampling steps. Overall, the relationship between the calculated and simulated random chance demonstrates that the algorithm for the random chance scenario worked well.

Fig 2 shows that in the random chance scenario, a low mean probability of observing codes leads to over 4000 sampling steps to reach theoretical saturation, regardless of the number of codes. As the mean probability of observing codes increases, the number of sampling steps declines rapidly with a decreasing trend to below 10 for all number of codes. This implies that mean probability of observing codes is more important than the number of codes for reaching theoretical saturation. The figure also shows that both purposive scenarios are more efficient than random chance. For a low mean probability of observing codes, the differences between scenarios are the largest. With the random chance scenario, 101 codes in the population and a mean probability of observing codes that is smaller 0.1, it generally requires more than 1000 sampling steps to reach theoretical saturation in 95% of the cases. Under the same conditions, this number is reduced to about 46 information sources in the minimal information scenario and to about 20 in the maximal information scenario. As the mean probability of observing codes becomes larger, the random chance and minimal information scenarios require about the same number of sampling steps for theoretical saturation, while the maximal information scenario requires less. Notable is that the numbers of both purposive scenarios fall within the range of common indications of sample size from the literature (below 50). Our result confirms that this indication is not far from accurate. Finally, in the maximal information scenario, the number of sampling steps for reaching theoretical saturation has little variance for different values of the mean probability of observing codes. This is because the high level of efficiency gives little room for variation.

**Fig 3:**
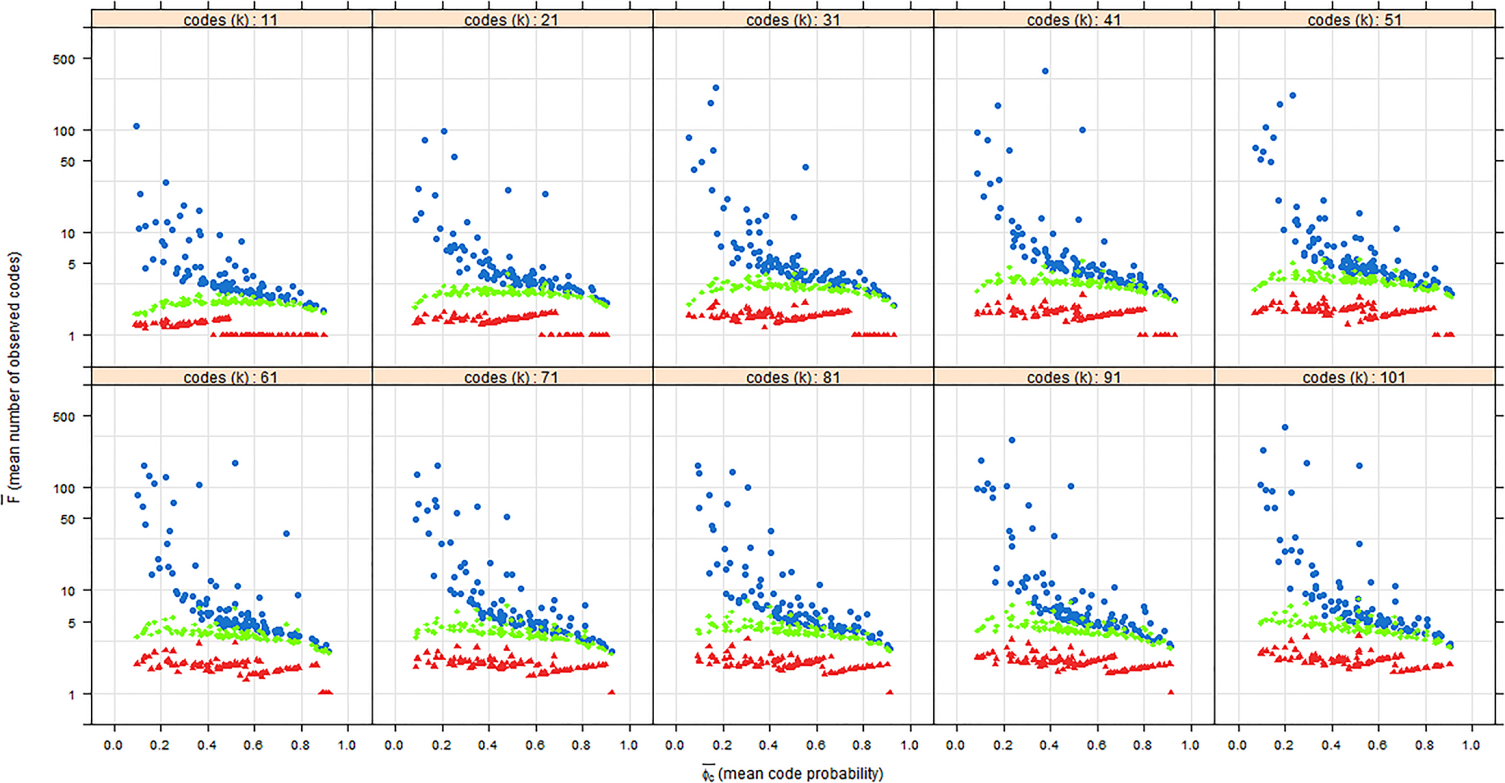
$\bar{F}$ upon reaching theoretical saturation against $\bar{\Phi_{c}}$ for the values of *k* between 11 and 101. The blue dots represent random chance, the green diamonds represent minimal information, and the red triangles represent maximal information.

Fig 3 plots the mean number of observations per code $\left(\bar{F}\right)$ upon reaching theoretical saturation for each scenario against the mean probability of observing codes for different values of the number of codes. Again, the dots represent random chance, the diamonds represent minimal information, and the triangles represent maximal information.

In line with the result above, the mean probability of observing codes has a greater influence than the number of codes on the mean number of observations per code in the random chance scenario. Second, the random chance scenario gives the largest number of repetitive codes (over 400) at a low mean probabilities of observing codes. This is explained by the fact that this scenario has the most sampling steps on average. However, for higher mean probabilities of observing codes, the random chance scenario yields about the same number of codes as minimal information, which is between 3 and 5. Finally, the maximal information scenario only yields between 1 and 3 observations per code. This low number of codes makes the use of repetitive codes for maximal information very limited.

Overall, the results show that there is a clear trade-off between the efficiency of the scenario and the number of repetitive codes. To increase the credibility of one’s research, it is possible to aim for a minimum number of observations of each code *(ν)*. For reasons of space, I do not simulate the various scenarios for different minimum numbers of observations of each code, but a calculation (see S1 Appendix Section D: Repetitive codes: F14) reveals that it is relatively easy to aim for observing codes multiple times. On average, to obtain a repetition of one (*ν* = 2) based on the calculated random chance, 2.3 extra sampling steps are required, which is an increase of about 10%. For *ν* = 3, 3.66 extra steps are required (about 17%), and for *ν* = 4, 4.66 extra steps are required (about 21%). The number of extra steps required is even smaller for both purposive scenarios as these are more efficient.

## Conclusions

The results for the purposive scenarios produced the same range of minimum sample sizes (below 50 information sources) as tentatively indicated in the literature. The simulations also uncovered mechanisms that give key insights into the estimation of the minimum size of a qualitative sample. The mean probability of observing codes is more important than the number of codes in the population for reaching theoretical saturation. Furthermore, when the probability of observing codes is low, the purposive scenarios are much more efficient than the random chance scenario. When this probability is high, the differences between scenarios are small. Finally, the more efficient a scenario is, the lower the mean number of observations per code, but only a few sampling steps are required to increase the minimum number of observations of all the codes.

### Limitations and further research

This paper has two potential limitations that deserve discussion. First, critics could claim that the scenarios are mechanistic and do not represent real-world sampling procedures. I used ideal typical scenarios that capture the full range of possible empirical sampling procedures. Researchers who view their research through the lens of these scenarios are likely to observe that their sampling procedure shares characteristics with at least one of the three scenarios or that their sampling procedure is a mixture of two scenarios. Future researchers can also simulate other scenarios that they conceive and even include different sampling strategies in their simulations, like snowball sampling or sampling for maximal variation [12,13].

Second, I simulated a broad range of scenarios for the purpose of this paper, but other simulations are also possible. For example, I simulated only one population per combination of mean probabilities of observing codes and the number of codes. This lack of variation could cast doubt on the robustness of my results. However, there was a large variation among the 1100 populations, as the number of codes was not important for the minimum sample size and because the variance around the mean probability of observing codes was not important. By letting the mean probability of observing codes vary between 0.09 and 0.91, I only considered a range of probabilities that is realistic in an empirical setting. I also did not vary the population sizes. Instead, I chose a large number that produced conservative estimates of the minimal sample size. It would be empirically interesting to vary the sample sizes in the simulations. For computational reasons and to reduce the complexity of this paper, I left this challenge for future researchers. Finally, I did not simulate different minimum observations per code, as the formula based on random chance gave sufficient insights into this issue.

### Guidelines for purposive sampling

Based on these insights, I formulate a set of guidelines for sampling in qualitative research. I am aware that such guidelines are contested by many qualitative researchers, but Tracy [33] rightfully argues that criteria or guidelines are useful to represent the core of the craft of doing research, and help improve quality. Some of these guidelines are already implemented by many scholars, but for completeness I mention them here. The guidelines are not intended as formal mechanistic rules, but rather as an aid to making informed choices about the sampling and how to report it.

The guidelines for sampling in qualitative research are as follows:

1. **Identify a population of information sources and sub-populations**. This does not need to be a formal sampling frame, but the researcher does need to sketch the kind of information sources that exist in the population and whether there are sub-populations. If there are sub-populations, the researcher can argue:
  - The basis for distinguishing sub-populations.
  - Whether the sources are interchangeable in a sub-population.
  - Whether the sub-populations serve a comparative purpose or are used for other means.
  - The process of data collection, sampling, and analysis per sub-population.
  - Other criteria that are deemed important by the researcher.
  The more detailed the researcher’s description of the population and sub-populations, the better. This is especially true when the researcher aims to use a maximal information scenario. However, as the researcher usually keeps an eye open for new developments, the delineation of the population and sub-populations can be updated at each sampling step.
2. **Estimate an order of magnitude of the number of codes per sub-population**. This estimation is based on:
  - The complexity and scope of the research question.
  - The existing theory and information available about the sub-population.
  - Other possible factors that are deemed to be of influence.
  Because the influence of the number of codes on theoretical saturation is small, it is more important to give an order of magnitude than an exact number. The estimation can be adapted after each sampling step.
3. **Estimate the mean probability of a code being observed**. The researcher does not need to know what a reasonable probability is at the start of the research, but it is likely that after consulting a number of information sources, the researcher will have enough information to make the assessment. The judgment at least depends on:
  - The likelihood of an information source actually containing codes (is required information rare in the population and what are the chances of non-response?).
  - The willingness and ability of the source (or its authors) to let the code be uncovered (are there strategic interests?).
  - The probability that the researcher is able to observe the code (based on the researcher’s prior research experience and familiarity with the topic).
  - Other criteria that are deemed important by the researcher.
4. **Assess which scenario is most applicable to each sub-population**.
  - Random chance is only appropriate if after a substantial number of sampling steps, the researcher still has little or no idea about the characteristics of the sub-population and where codes can be found. In that case, random chance serves as a fallback scenario. If theoretical saturation is reached under random chance, then it is also reached in the other two scenarios. With conservative estimates of the mean probability of observing codes, the minimum sample size is over 4000 information sources, while for higher means, the minimum sample size rapidly drops to below 100 at probabilities of around 0.3 and below 50 at probabilities of 0.4.
  - Choosing a minimal information scenario requires some argumentation. Most important is that the researcher makes it plausible that a new code will be observed at each sampling step. This is something that the researcher will experience as the research progresses. If at a sampling step an information source does not yield any new codes, the researcher can opt for increasing the number of sampling steps by one. Usually there is little need to aim deliberately for multiple observations per code, because the scenario delivers sufficient repetitive codes. Under low estimates of the mean probability of observing codes, the minimum sample size for minimal information is around 50, while for higher means the minimum sample size is below 25.
  - The researcher can only choose maximum information when there is already a full overview of all the information sources in the (sub-)population and how information-rich these sources are (e.g. how many codes they contain). However, as maximum information makes very strong assumptions, the choice needs proper argumentation. The benefit of the maximum information scenario is that even under low estimates of the mean probability of observing codes, the minimum sample size is only 20 information sources. For higher means, the minimum sample size drops below 10. However, unless there is already strong theory present, I advise to aim for multiple observations of each code to guard against misinformation.
  It is unlikely that a scenario will be followed exactly; rather, the researcher will notice that the sampling procedure falls somewhere in between the scenarios. As such, the researcher can argue which scenario the sampling procedure resembles most. The researcher can use the results from the simulations above to assess whether theoretical saturation is likely to have been reached.
5. **Choose a fitting sampling strategy**. The researcher should take into account that the sampling strategy (see [12,13]) needs to lead to a sufficiently broad reach across information sources in the population to be able to cover all the codes relevant to answering the research question.
6. **Account for these steps when reporting the research**. State why a scenario, with its associated minimum sample size is appropriate. The researcher can choose to report the total number of unique codes observed after a given number of sampling steps (for example: 4–5). This can help assess the plausibility of the scenario. The researcher can further report the number of times each code was observed and whether there were reasons to suspect that some codes were not credible. Finally, the researcher can assess if theoretical saturation at the population level was reached.

Following these recommendations does not mean that overall quality of the research is good. The recommendations can only help to improve the sampling, which is but one aspect of the entire process. In addition, in many instances, codes are not yet fixed at the start of the research. Rather, they become more known as the research progresses. I suggest that researchers reevaluate their assessment during each sampling step.

Keeping the analyses in mind, I recommend that researchers should generally opt for a minimal information strategy, as it makes reasonable assumptions, it is efficient, and it yields sufficient codes. Whether saturation has been reached remains in the argumentative judgment of the researcher. These guidelines can aid the researcher in making this judgment and the readers in assessing it. Overall, the results and the guidelines offered in this paper can improve the quality and transparency of purposive sampling procedures. Therefore, I encourage fellow researchers to consider using these ideas and guidelines and to improve upon them where they see fit.

## Supporting information

S1 Appendix Technical details. Mathematical details of the simulation.
(DOCX)

S1 File R-code for the simulations. Code for the simulations in R.
(R) Click here for additional data file.

S2 File Simulated data. The simulated data set used for this study.
(CSV)
