# Supplementary material for: (I Can’t Get No) Saturation: A simulation and guidelines for sample sizes in qualitative research
Source: PLoS One. 2017 Jul 26;12(7):e0181689. doi: 10.1371/journal.pone.0181689 (PMC5528901; doi:10.1371/journal.pone.0181689)
Supplement: S1 Appendix — Mathematical details of the simulation. (DOCX) [file pone.0181689.s001.docx]

**S1 Appendix: Technical details**

**Section A. Definitions**

I denote the population by *J* and a given information source by *i.* The number of sampling steps is given by *n*, and the number of sampling steps required for theoretical saturation is given by *n_s_*. Moreover, I denote a sub-population by *j* and the number of sub-populations by *m*. When working at the sub-population level, everything needs to be subscripted with *j*. However, for convenience of notation, I work here at the population level.

The number of codes in the population is denoted by k. The vector of codes in the population is given by *C,* which has length k:

1. $C=(C_{1},C_{2},\ldots,C_{k})$

In the case that the vector *C* represents all the codes in the population, it is subscripted with *J*. By definition, the vector *C_J_* has length *k* and all the values are 1 (all the codes are present, but still unobserved). In addition, each information source *i* in the population consists of a vector of codes denoted by *c_i_* that is also of length *k*. It indicates whether a code is present (1) or absent (0) in a source:

1. $c_{i}=(c_{i1},c_{i2},\ldots,c_{ik})$

The vector *C_fn_* is also of length *k* and contains how often (e.g. the frequency) each code has been observed after *n* sampling steps. The researcher can use the vector *C_fn_* to assess the credibility of the research by looking at rare codes. It is easily obtained by calculating the sums of all vectors *c_i_*.

1. $C_{fn}= \sum_{i=1}^{n} c_{i}$

The mean of *C_fn_* is denoted by $\bar{C_{fn}}$; at n_s_ it is denoted by $\bar{C_{{fn}_{s}}}$. Further, I denote a vector *C_On_*, also of length *k*, that expresses which codes have been observed after *n* sampling steps. It is obtained by substituting all the values of *C_fn_*>0 with 1. Theoretical saturation is reached if:

1. $C_{J}=C_{On}$

**Section B: Mean probability of observing codes**

The presence or absence of an example code *c_1_* in an information source *i* can be seen as a random Bernoulli trial. The probability that *c_1_* is present (1) is given by Φ*_c1_* and the probability that it is absent (0) is given by 1-Φ*_c1_*. The vector Φ*_c_* of length k contains the probabilities of each code being present:

1. $\Phi_{c}=(\Phi_{c1},\Phi_{c2}\ldots,\Phi_{\mathrm{ck}})$

The vector *Φ_c_* can be described by a beta distribution, which has two parameters that can only take a value larger than 0: *α* and β. The *expected mean* of the distribution is given by:

1. $E[\Phi_{c}]=\frac{\alpha}{\alpha+\beta}$

The variance of the beta distribution lies between 0 (all the values are the same) and 0.25 (exactly half of the values are 0 and half of the values are 1). The expected variance is given by:

1. $Var\left[ \Phi_{c} \right]=\frac{\alpha\beta}{\left( \alpha+\beta\right)^{2}(\alpha+\beta+1)}$

**Section C: Reaching theoretical saturation**

The expected mean of the distribution is a theoretical construct that I use in my simulations. However, qualitative researchers usually deal with the observed distribution of probabilities, which I will denote as $\bar{\Phi_{c}}$. It is obtained by calculating the mean of vector Φ*_c_*. *E[*Φ_c_*]* and $\bar{\Phi_{c}}$ are expected to have a high – but not perfect – correlation, because observed (empirical) distributions always contain deviations that result from random chance.

To answer the research question, I need to know the value of *n* for *F4* to be true with a certain probability, which I denote by *p_n_.* It can be seen as the chance that theoretical saturation has been reached after *n* sampling steps. The larger the value of *p_n_*, the larger *n* needs to be, given *k* and $\bar{\Phi_{c}}$. Consistent with the conventions in quantitative research, I choose to set *p_n_* at 0.95. This means that I look for a value of *n* for which all the codes are observed in 95% of the times that I draw a sample. The value of 95% is arbitrary, but it gives a degree of certainty that is equal to the common significance threshold in the social sciences.

In the case of random chance, the probability *p_nc1_* that example code *c_1_* is actually observed at least once after *n* sampling steps is equal to 1 minus the probability that the code has not been observed after *n* sampling steps:

1. $p_{nc1}=1-{(1-\Phi_{c1})}^{n}$

In the case of *k* codes, the probability *p_n_* of observing all the codes is the product of each probability from *F8*:

1. $p_{n}=\prod_{c=1}^{k} (1-\left( 1-\Phi_{ck} \right)^{n})$

A special condition occurs when all the values of *Φ_c_* are equal, which implies a variance of zero. The larger the values of *α* and/or *β*, the more this condition is approximated. The value of Φ*_c_* can then be denoted as a single number Φ*_k_*. Though not very realistic, this special condition is mathematically convenient, since it is the only case in which we can calculate *n_s_* directly. This allows me to explore the relationship between *n_s_*, *k*, and Φ analytically under the conditions of random chance. First, I can simplify *F9* to:

1. $p_{n}={(1-\left( 1-\Phi_{k} \right)^{n})}^{k}$

This can be rewritten in terms of *n*. Moreover, I want to obtain the value of *n_s_*, which is a specific value of *n*:

1. $n_{s}= \frac{\ln(1-\sqrt[k]{p_{n}})}{ln(1-\Phi_{k})}$

This formula gives the number of sampling steps needed to reach theoretical saturation in 100**p_n_*% of the cases, based on random chance, as a function of *k* and *Φ_k_*. If *p_n_* is set at 0.95, then the number of required sampling steps is formally denoted as *n_s(0.95)_*. However, since *p_n_* is fixed throughout this paper at 0.95, the addition of the number has little added value. For convenience of notation, I continue to refer to *n_s_.*

If the values of Φ*_c_* are not equal, it is not possible to write *F9* in terms of *n_s_*, which means that the minimum sample size cannot be calculated mathematically. In that case, simulation techniques can be used to approximate the answer [41].

**Section D: Repetitive codes**

To enhance the credibility of the research, it is also possible to aim deliberately for a minimum number of observations of each code. This minimum is denoted by *ν* (Greek letter nu). A value of *ν*=2 means that each code is observed at least twice and thus that each code is repeated once. The probability of observing *k* codes, with probability Φ*_ck_*, for a *ν* number of times after *n* sampling steps is given by:

1. $p_{n}={(\prod_{k=1} 1-\left( 1-\Phi_{ck} \right)^{n})}^{\nu}$

In the specific condition that all the values of Φ*_c_* are equal, this simplifies to:

1. $p_{n}={{(1-\left( 1-\Phi_{k} \right)^{n})}^{k})}^{\nu}$

Rearranging and replacing *n* with *n_s_* gives:

1. $n_{s}= \frac{\ln(1-\sqrt[k\nu]{p_{n}})}{ln(1-\Phi_{k})}$

*F14* provides, based on random chance, the minimum number of sampling steps to observe each code *ν* times, conditional on the other variables. Again, in the case that the values of Φ*_c_* are not equal, it is not possible to write *F12* in terms of *n_s_*.

**Section E: From sub-population to population**

Finally, if these principles are applied to *m* sub-populations, the probability of reaching theoretical saturation at the population level is calculated by:

1. $p_{n}=\prod_{m=1} p_{n_{j}}$

In the case that all the probabilities of reaching theoretical saturation are set as equal for all the sub-populations, *F15* simplifies to:

1. $p_{n}={{(p}_{n_{j}})}^{m}$

If there are three sub-populations and $p_{n_{j}}$ in each of these is set at 0.95, then the probability of reaching theoretical saturation in the overall population *p_n_* is 0.857. However, unless the purpose is to compare sub-populations, this number should be considered as a minimum probability. Since sub-populations can have an information overlap, codes that have not yet been observed in one sub-population may have already been observed in another.

**Section F: Simulation**

Using the *F6*, I simulate $\bar{\Phi_{c}}$. for all discrete combinations of *α* and *β* that lie between 1 and 10, which leads to probabilities that vary between 1/11 (~0.09) and 10/11 (~0.91). Each information source in a population is represented by a vector *c_i_*.

The size of each population is 5000 information sources, which means that each population is actually a 5000 by *k* matrix, with only values of 0 and 1. I choose a population size of 5000 to prevent this number from influencing the results.^^[[1]](#footnote-1)^^ For smaller populations, theoretical saturation is likely to be reached earlier, but I take a conservative approach. Further, in line with my earlier argument about interchangeability of information sources, I impose a condition whereby each of the *k* codes is actually present in at least two information sources in the population.

By setting the *α* and *β* parameters to >1, I obtain only unimodal distributions and exclude all U-shaped or J-shaped distributions. These shapes imply that within one distribution, the probability of observing codes that are rare or common is larger than the probabilities of any other code being observed. U-shaped or J-shaped distributions are not impossible but less plausible than a distribution with only increasing or decreasing probabilities.

All three scenarios operate in a similar manner. After generating a population, an information source is selected:

- *Random chance* selects information sources based on probability.
- *Minimal information* works in the same way as random chance, but adds an extra condition that *at least* one new code must be observed per sampling step. Otherwise it is discarded.
- *Maximal information* first identifies a set of information sources that contain the largest number of unobserved codes. From this set, which often consists of a small number of information sources, it randomly selects an information source.

If the source has not been selected before, it is added to the sample. After each sampling step, the vector *C_On_* is updated with the new codes that are found in *c_i_*. Next, *C_On_* is compared with *C_J_*; if the two are equal, then theoretical saturation is reached in the population. If so, the process stops and *n_s_* is reported. Otherwise, the next sampling step takes place and a new information source is selected from the population.

I apply each of the three sampling scenarios to each population 500 times. This produces a distribution for each scenario with values of *n_s_*. From this distribution, I obtain *n_s_*. Moreover, for each population, I obtain the mean value over 500 simulations of $\bar{C_{{fn}_{s}}}$ (the vector containing the frequencies of observed codes) as an indicator of repetitive codes. For convenience of notation, *F* represents a vector of length 500 containing the values of $\bar{C_{{fn}_{s}}}$. The mean of this vector is given by $\bar{F}$: it is equal to the more elaborate notation $\bar{\bar{C_{{fn}_{s}}}}$ that I shall not use further.

Moreover, for each population, I obtain the mean value over 500 simulations of $\bar{C_{{fn}_{s}}}$ (the vector containing the frequencies of observed codes) as an indicator of repetitive codes. For convenience of notation, *F* represents a vector of length 500 containing the values of $\bar{C_{{fn}_{s}}}$. The mean of this vector is given by $\bar{F}$: it is equal to the more elaborate notation $\bar{\bar{C_{{fn}_{s}}}}$ that I shall not use further.

1. Technically, for the random chance and minimum information scenarios, there is no need to specify a population size a priori. One can just generate a new information source at each step. However, maximal information does require an a priori specified population, as it assumes that the researcher has full knowledge of the population. To keep the results comparable across scenarios, I also specify a population size for random chance and minimum information. [↑](#footnote-ref-1)
